# Genetic and Pathogenic Characteristics of Variant Avian Reovirus Strains Isolated from Diseased Chickens in China

**DOI:** 10.3390/microorganisms13112450

**Published:** 2025-10-25

**Authors:** Shiqi Niu, Zihua Wu, Shenghui Pan, Tianxin Ma, Yunxiang Zhang, Bangfeng Xu, Dawei Yan, Qiaoyang Teng, Chunxiu Yuan, Xue Pan, Zhifei Zhang, Minghao Yan, Xiaona Shi, Zejun Li, Qinfang Liu

**Affiliations:** Shanghai Veterinary Research Institute, Chinese Academy of Agricultural Sciences, Shanghai 200241, China; 15837283120@163.com (S.N.); wuzihua6@163.com (Z.W.); panshenghui37@163.com (S.P.); matianxin12@163.com (T.M.); 13507656505@163.com (Y.Z.); xubangfeng@163.com (B.X.); yandawei@shvri.ac.cn (D.Y.); tengqy@shvri.ac.cn (Q.T.); yuanchx@shvri.ac.cn (C.Y.); panxue@shvri.ac.cn (X.P.); nzhangzhifei@163.com (Z.Z.); m17519478956@163.com (M.Y.); shixiaonare@163.com (X.S.)

**Keywords:** avian reovirus, arthritis, pathogenicity, phylogenetic analysis

## Abstract

Avian viral arthritis (AVA), caused by avian reovirus (ARV), is a viral disease in chickens that has led to significant economic losses in the poultry industry. Recent studies have shown that traditional ARV vaccines based on the S1133 strain fail to protect against emerging ARV variants. In this study, we isolated and characterized three ARV strains (G4, YV, WF) from immunized chicken flocks with respiratory and arthritic symptoms. Genomic analysis revealed that the σC genes of G4, YV, and WF shared only 55.5%, 55.7%, and 58.7% sequence homology, respectively, with the S1133 strain. Phylogenetic analysis placed them in different branches, indicating they are variant strains. YV and WF belong to genotype III, and G4 falls into genotype VI. Whole genome analysis revealed gene segment reassortment among the variants. Pathogenicity testing in three-week-old SPF chickens showed that G4 (genotype VI) caused swelling of footpads, whereas WF (genotype III) did not. G4-infected chickens exhibited significantly higher viral loads in the thymus, lungs, spleen, and bursa of Fabricius than those in the WF-infected chickens, indicating viruses from different genotypes showed various pathogenesis. These results suggested an urgent need for new updates of vaccines against the variant ARVs, especially the genotype VI virus.

## 1. Introduction

Avian reovirus (ARV) was first isolated from broilers with chronic respiratory disease by Fahey and Crawley in 1954 [1]. ARV belongs to the Reoviridae family and is a member of the Orthoreovirus genus [2]. The ARV virion is a non-vesicular membrane icosahedral particle with a diameter of approximately 75 nm and has a double-layered nucleocapsid structure [3]. Susceptibility to ARV infection is age-related, with chicks being the most vulnerable and resistance increasing rapidly as birds mature [4]. ARV infection causes viral arthritis or tenosynovitis [5], enteric disease [6] and malabsorption syndrome [7] in chickens, which are characterized by stunting, high mortality rates [8], and secondary immunosuppression, posing a significant threat to the avian industry worldwide [9].

The genome of ARV consists of segmented, double-stranded RNA, with genomic segments that are classified into three classes based on their electrophoretic rate sizes: L (large, L1, L2, L3), M (medium, M1, M2, M3), and S (small, S1, S2, S3, S4) segments [10]. The ARV genome expresses at least 12 primary translation products, consisting of eight structural proteins and four nonstructural proteins [11]. The outer capsid protein of the virus, σC protein, is encoded by the S1 gene and serves as a cell attachment protein [12]. The σC gene evolves at a higher rate than the other structural proteins, and the ARV has been divided into six different lineages according to the σC gene [13].

The ARV virus was identified as an avian reovirus in 1967 [14] and subsequently broke out in Hungary [15], Tunisia [16], Brazil [17], the United States [18], Japan [19], and Australia [20]. Vaccination is the most effective method to prevent the disease. The commercially available vaccines based on S1133-like strains were developed in the late 1970s and early 1980s. However, the S1133 strain belongs to genotype 1. Recent studies have shown that S1133-like vaccines cannot provide adequate protection against the novel variant ARVs as a consequence of frequent variation in ARV [18]. The ARV variants and novel strains have been reported in the US since 2011 [18,21,22,23], which have caused significant economic losses to the poultry industry in the US [24,25]. Lu et al. reported that 80% of ARV isolated in the US belong to the novel ARV variant genotypes 2, 3, 4, 5, and 6 [18]. The ARV was first isolated from diseased chickens in China in 1985. Recently, the novel variant ARVs have been isolated from S1133 vaccinated chickens [26,27].

In this study, three variant ARVs, named YV, WF, and G4, were isolated from clinically diseased chickens in China. As indicated by genetic evolution analysis, they belong to genotypes III (YV, WF) and VI (G4), respectively. Genomic sequence alignment and recombination analysis revealed the genetic variations and rearrangements among the three strains. Pathogenicity assessments of the isolates were conducted on 21-day-old SPF chickens. The genotype VI G4 replicated efficiently in the thymus, lung, spleen, and bursa of Fabricius of the infected chickens and caused swelling on footpads. While the genotype III WF did not cause swelling on footpads and the viral loads in the thymus, lung, spleen, and bursa of Fabricius were lower than those of G4, suggesting that the different genotype ARV variants showed various virulence in chickens.

## 2. Materials and Methods

### 2.1. Cells, Animals, and Reagents

Primary cultures of chicken embryo fibroblast (CEF) cells were prepared from 9-day-old SPF embryos using standard tissue culture techniques. Cells were grown in Dulbecco’s modified Eagle’s medium (DMEM) (Hyclone, Logan, UT, USA), supplemented with 10% fetal bovine serum (FBS; Biowest, South American origin, Riverside, MO, USA), 100 U/mL penicillin, and 100 μg/mL streptomycin (Sangon Biotech (Shanghai) Co., Ltd., Shanghai, China) at 37 °C, with 5% CO_2_. Leghorn male hepatocellular (LMH) cells were purchased from the American Type Culture Collection (ATCC, Manassas, VA, USA) and cultured in Dulbecco’s Modified Eagle Medium: F12 (DMEM/F12) (Hyclone, Logan, UT, USA), supplemented with 10% FBS, 100 U/mL penicillin, and 100 μg/mL streptomycin at 37 °C, with 5% CO_2_ for routine maintenance. Specific pathogen-free (SPF) chicken embryos and chickens were purchased from Zhejiang Lihua Agricultural Technology Co., Ltd. (Ningbo, China).

### 2.2. Clinical Samples and Virus Isolation

The tendons, joints, heart, liver, spleen, and lungs were collected from diseased chickens with respiratory symptoms and arthritis on three vaccinated chicken farms in eastern China between 2017 and 2019. One gram of tissue was homogenized in 1 mL of phosphate-buffered saline (PBS) supplemented with 1000 U/mL penicillin and 1 mg/mL streptomycin. The tissue homogenate was centrifuged at 5000× *g* for 10 min at 4 °C The supernatant was inoculated onto CEF cells in a T25 flask. The flasks were incubated at 37 °C with 5% CO_2_ for one week. The cytopathic effect (CPE) was observed daily in comparison to negative control cells. Cells that did not appear CPE after one week were frozen at −80 °C, thawed at room temperature three times, and then re-incubated with fresh LMH cells for passaging. Cells that appeared with 50% CPE were frozen and thawed three times and then centrifuged to collect the supernatants, which is stored at −80 °C. The virus titer was determined using LMH cells, and the Tissue Culture Infectious Dose 50 (TCID_50_) was calculated using the Reed-Muench method [28]. To identify the isolated viruses, the total RNA in original samples and cell culture was extracted using TIANamp Virus RNA Kit (Tiangen Biotec Co., Ltd., Beijing, China) according to the manufacturer’s instructions. Viral cDNA was generated from genomic RNA using M-MLV Reverse Transcriptase, following the manufacturer’s protocol (Vazyme, Nanjing, China).

### 2.3. Genomic Sequencing of the Isolated ARV Strains

The PCR reaction mixture contained 25 μL of 2× Taq Plus Master Mix II (Vazyme, Nanjing, China), 2 μL of cDNA, 2 μL of each 10 μM primer (Supplementary Table S1), and 19 μL of double-distilled water (ddH_2_O). PCR amplification was performed using the following cycling conditions: initial denaturation at 95 °C for 3 min; 35 cycles of denaturation at 95 °C for 15 s, annealing at 56 °C for 15 s, and extension at 72 °C for 60 s per 1000 bp; followed by a final extension at 72 °C for 10 min and storage at 4 °C. PCR products were separated by electrophoresis on a 1% agarose gel (Thermo Fisher Scientific, Waltham, MA, USA) at 120 V for 15 min and visualized using a gel documentation system. The ten genomic fragments of the ARV isolate were cloned into the pMD18-T vector and submitted to Shanghai Qingke Biotechnology Co., Ltd. (Shanghai, China) for Sanger sequencing [29]. The sequences were deposited into GenBank to obtain accession numbers. The ORFs, amino acid translation, sequence alignment, and pairwise sequence of ARV isolates were aligned using SnapGene 4.2.4. Phylogenetic analysis of the genomic segments of L-, M-, and S-class genes and σC amino acid sequences was performed using MEGA 6, and phylogenetic trees of all the gene segments were constructed using the neighbor-joining method with a bootstrap value of 1000 replications [30]. The nucleotide and amino acid sequence homology of the σC protein from ARV isolates with the vaccine strain S1133 was analyzed using the BLAST online platform (NCBI BLAST+ version 2.16.0, https://blast.ncbi.nlm.nih.gov/Blast.cgi, accessed on 1 August 2024).

### 2.4. Pathogenicity of the ARV Isolates in SPF Chicken

To evaluate the pathogenicity of ARV isolates in chickens, eighteen three-week-old SPF chickens were randomly divided into three groups (each *n* = 6) for challenge. Each chicken in the challenge groups was inoculated with 200 μL of virus containing 2 × 10^5^ TCID_50_ of genotype III (WF strain) and VI (G4 strain) by footpad puncture, and a negative control group was inoculated with the same amount of sterile PBS. The chickens in each group were housed in separate isolators with negative pressure and observed continuously for five days post infection. Clinical signs of viral tenosynovitis and arthritis were observed and recorded, and the infected chickens were euthanized at 3 and 5 days post-infection (dpi). Tissue samples from the tendon, thymus, lung, spleen, and bursa of Fabricius were collected, and a portion of each was used for viral titration on LMH cells. To preserve tissue structure, the remaining samples were fixed in 4% paraformaldehyde for subsequent analysis. The fixed tissues were then embedded in paraffin and stained with hematoxylin and eosin (H&E) as previously described [31].

### 2.5. Statistical Analysis

Statistical analyses of viral titer data were performed using GraphPad Prism version 8.2 (GraphPad Software, San Diego, CA, USA). Two-way ANOVA followed by Tukey’s HSD post hoc test was applied, with *p* < 0.05 considered statistically significant. The sample size (*n* = 3 per group) was determined from pilot data and power analysis (α = 0.05, power = 0.8), which demonstrated effect sizes >3.9 (Cohen’s d) and statistical power exceeding 99% for major immune organs.

## 3. Results

### 3.1. Virus Isolation and Identification

Ten chickens were collected from each of three farms exhibiting respiratory and arthritic clinical signs. The reovirus-positive rates among these chickens ranged from 20% to 30%. Three ARV isolates, designated as YV, WF, and G4 strains, were successfully isolated from tissue samples using CEF cells. The LMH cells inoculated with the virus showed clear CPE at 24 h p.i., manifested by cell fusion to form syncytia of varying sizes. After plaque assay purification for three times, the three ARV isolates were propagated and titrated on LMH cells. After virus purification, the titers for YV, WF, G4 strains were 10^6.5^ TCID_50_, 10^6.5^ TCID_50_, and 10^6^ TCID_50_ per 0.1 mL in LMH cells, respectively.

### 3.2. Sequencing and Analysis of Complete Genomes of the Three ARVs

**Figure 1:**
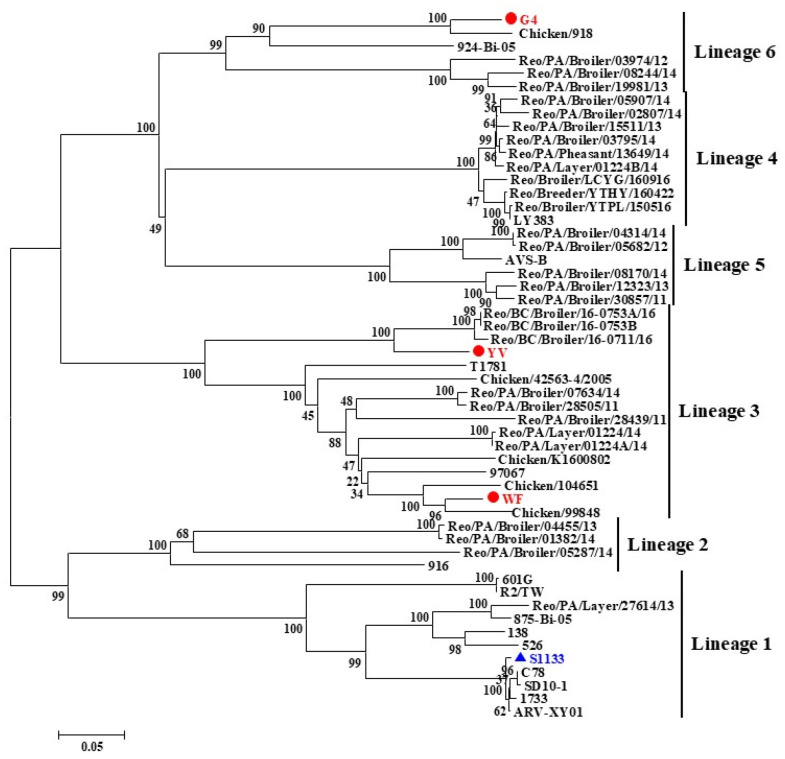
Phylogenetic tree of ARV variant strains based on the σC gene sequence. The isolated strains clustered into six genotypic groups. The present isolates belong to genotypes III (YV and WF) and VI (G4). The phylogenetic tree was constructed using the neighbor-joining method with the Jukes–Cantor genetic distance model. Isolates are marked with red circles, the vaccine strain with a blue triangle, and the scale bar indicates genetic distance.

The complete sequences of the 10 genomic segments of the three ARVs were obtained through sequencing and uploaded to GenBank (GenBank accession numbers: PV158362–PV158391). The open reading frame (ORF) of the 10 genomic segments of the isolates ranged from 1104 bp (S4) to 3882 bp (L1). The genomic characteristics of the isolates were consistent with the ARV reference strain, and all 10 segments were predicted to encode 12 putative proteins: λA, λB, λC, μA, μB, μNS, σA, σB, σNS, p10, p17, and σC. Segment S1 was identified as a tricistron segment, containing partially overlapped ORFs that encode p10 protein, p17 protein, and σC protein. The σC protein serves as the receptor binding protein of ARV and exhibits high genetic variability, making it a widely used marker for genotype classification. The phylogenetic tree constructed using the σC nucleotide sequence revealed six distinct clades. The σC genes of the three isolates are distributed across different clades. The YV and WF strains clustered within genotype III, while the G4 strain belongs to genotype VI (Figure 1). The σC gene of the three isolates showed low amino acid homology (48.6–52.6%) with the S1133 vaccine strain, suggesting that these isolates are variant ARV strains. Notably, even the YV and WF strains within genotype III share only 68% homology, highlighting the rapid evolutionary rate of the σC gene.

### 3.3. Phylogenetic Analysis of ARV Genes

**Figure 2:**
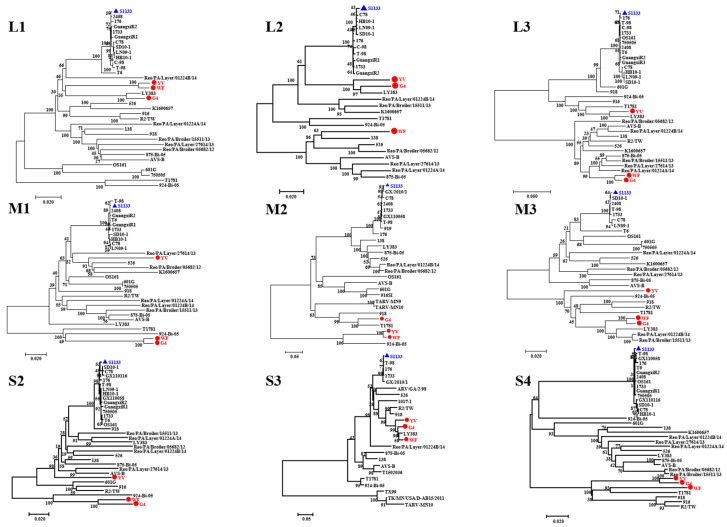
Phylogenetic trees based on the nucleotide sequences of the homologous L, M, and S genome segments of orthoreovirus species. Isolates are marked with red circles, the vaccine strain with a blue triangle, and the scale bar indicates genetic distance.

To determine the genetic evolutionary relationship among the three variant ARV strains, phylogenetic trees of the L-, three M-, and three S-class genes were constructed by using the neighbor-joining method (Figure 2). Overall, the L1, M3, S3, and S4 genes of the three isolates are located in the same clades, indicating a close evolutionary relationship among these genes, which showed a close relationship with the Chinese strain LY383, suggesting a potential domestic origin. In contrast, the L3, M1, and S2 genes of the YV strain exhibited evolutionary divergence from the other two strains. The L3, M1, and S2 genes of YV strain are clustered with American strains, while those of the WF and G4 strains are clustered with Hungarian strains, suggesting possible reassortment events with strains from other regions. Notably, the reassortment patterns of the L2 and M2 genes in the three isolates are highly complex, suggesting that these two genes are particularly prone to reassortment. In summary, reassortment among these genes may alter their pathogenicity and immunogenicity, potentially contributing to immunization failure.

### 3.4. Pathogenicity of the ARV Isolates in SPF Chickens

**Figure 3:**
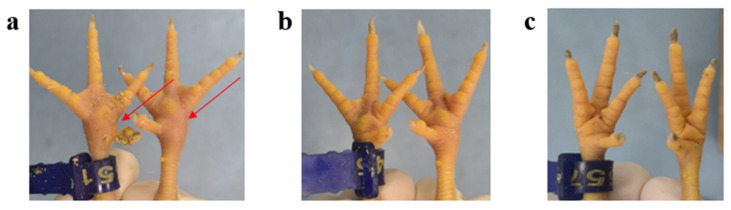
Clinical symptoms in SPF chickens following challenge with ARV isolates. (**a**) Footpad swelling (red arrows) in the G4-infected group. (**b**,**c**) Normal footpads in SPF chickens from the WF-infected and control groups.

**Figure 4:**
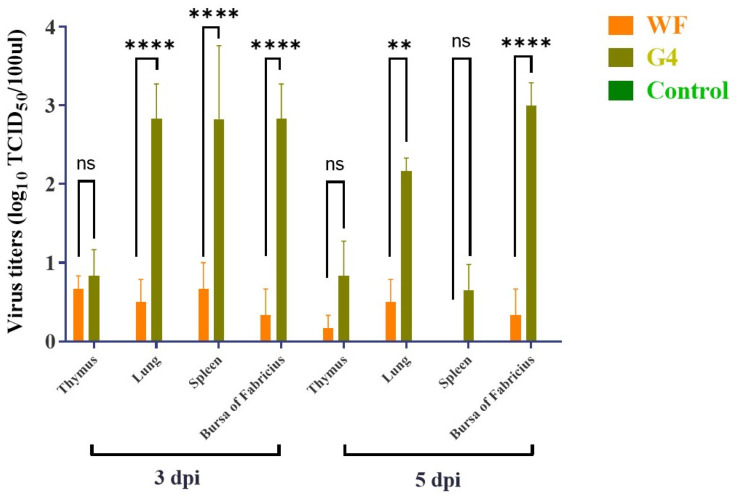
Viral titers in the thymus, lungs, spleen, and bursa of Fabricius. The WF-challenged, G4-challenged, and control groups are shown in different colors. The results indicate immune tissue tropism and potential respiratory tract damage caused by ARV infection. Statistical significance was determined by two-way ANOVA followed by Tukey’s HSD post hoc test (*p* < 0.05). Significant differences are indicated by asterisks: ns not significant, ** *p* < 0.01, **** *p* < 0.0001.

To investigate the pathogenicity of different genotypes of ARVs, genotypes III (WF) and VI (G4) were selected and tested on three-week-old SPF chickens. Throughout the experiment, the chickens in the control group remained asymptomatic and did not experience any fatalities. Notably, footpad swelling was most pronounced in the G4 challenge group, while chickens in the WF challenge group and control group exhibited normal footpads (Figure 3). Viral titers in dissected organs revealed viral replication in several immune organs, including the thymus, spleen, and bursa of Fabricius (Figure 4), suggesting a specific tropism of the ARV virus for immune tissues. Significant viral titers were also detected in the lungs, implying potential damage to the respiratory system. Additionally, viral loads in the G4 group were significantly higher than in the WF group, with the largest differences observed in the lung (mean difference: 2.334; 95% confidence interval (CI): 1.265–3.402; *p* < 0.0001), spleen (mean difference: 2.160; 95% CI: 1.092–3.228; *p* < 0.0001), and bursa of Fabricius (mean difference: 2.500; 95% CI: 1.432–3.568; *p* < 0.0001).

### 3.5. Histopathological Examination

**Figure 5:**
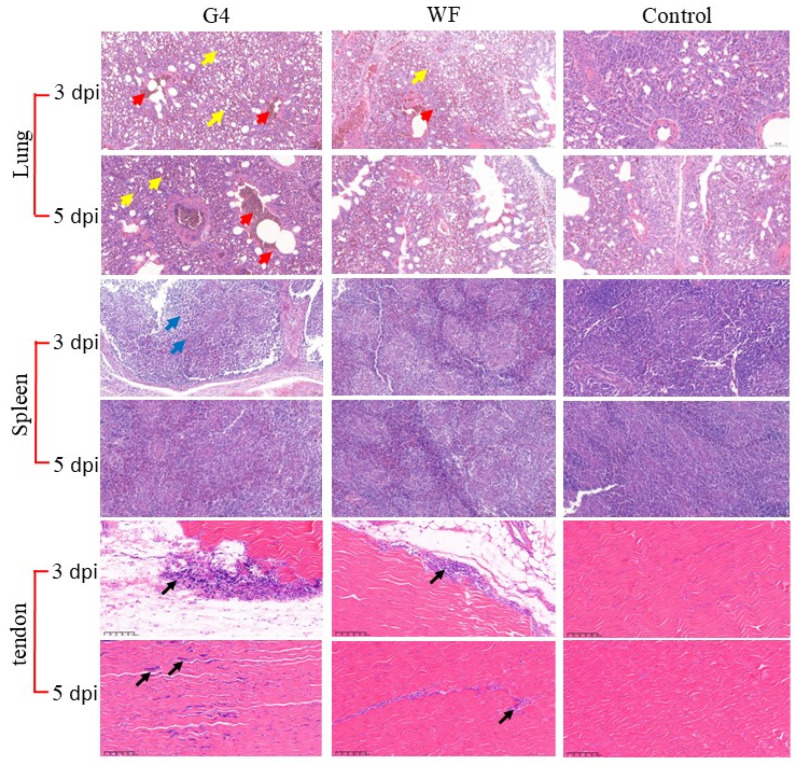
Histopathological examination (H&E staining) of chickens from challenged and control groups. In chickens infected with G4 and WF strains, lymphocytic infiltration was observed within the synovium, accompanied by localized inflammatory cell infiltration in the muscle fibers (black arrows). In the lungs, widespread vascular and capillary congestion was evident, with frequent peribronchial hemorrhage (yellow arrow) and marked accumulation of red blood cells within the lumen (red arrow). In the spleens of G4-challenged chickens, an indistinct corticomedullary structure and diffuse lymphocytic infiltration were noted (blue arrow). Magnification: 20×.

Chickens in the experimental and negative control groups were euthanized at 3 and 5 dpi for histopathological examination (Figure 5). In the tendon sections, lymphocytic infiltration was observed within the synovium, accompanied by localized inflammatory cell infiltration among muscle fibers. Lung sections revealed extensive vascular and capillary congestion, frequent parabronchial hemorrhage, and significant accumulation of red blood cells within the air spaces. Splenic sections showed an indistinct corticomedullary junction and a diffuse infiltrate of numerous lymphocytes. These findings indicate that the virus not only induces tenosynovitis characteristic of reovirus infection but also exerts substantial pathological effects on the immune organs and the respiratory system.

## 4. Discussion

ARVs have been clinically widespread since the first report in the United States in 1967 [32]; then, ARVs have been reported globally, including in Nata, Tunisia, Brazil, Iran, Japan, Korea, and other regions [16,17,33,34,35]. ARV infections can lead to respiratory symptoms, viral arthritis, and, in severe cases, mortality in chickens [27]. Given the increasing prevalence of ARV, field surveillance is vital to understand the prevalence of variant ARV in China [36]. This study provides significant insights into the genetic diversity and pathogenic characteristics of emerging variant avian reovirus (ARV) strains circulating in vaccinated chicken flocks in China. The isolation and characterization of the YV, WF, and G4 strains confirm the presence of antigenically distinct variants belonging to genotypes III and VI, posing a substantial challenge to current control strategies reliant on the S1133 (genotype I)-based vaccines.

σC protein is a primary cell attachment protein and a major target for neutralizing antibodies [37,38]. The low amino acid homology with the S1133 vaccine strain—48.6%, 51.1%, and 52.6% for G4, YV, and WF, respectively—provides a molecular explanation for the observed vaccine failures in the field flocks from which these strains were isolated. This level of divergence is consistent with reports of variant ARVs from other regions, such as the United States, where genotypes I–VI have become predominant [39]. Furthermore, even within genotype III, the σC sequences of the YV and WF strains exhibited only 68% homology, underscoring the remarkably high evolutionary rate of this gene and the continuous emergence of genetic diversity within established genotypes. This rapid evolution necessitates continuous genomic surveillance.

Whole-genome phylogenetic analysis revealed a complex reassortment for these ARV isolates [40]. The L1, M3, S3, and S4 of the three isolates showed close relationships with the Chinese strain LY383, suggesting potential domestic lineage origins. While the clustering of YV’s L3, M1, and S2 genes with American strains, contrasted with WF and G4 clustering with a Hungarian strain for these same segments, indicating reassortment events involving viruses of geographically distinct origins. The particularly complex reassortment patterns was observed in the L2 and M2 genes of the three isolates, which further highlight these segments as hotspots for genetic exchange. Reassortment is a critical mechanism for ARV evolution [41,42,43], potentially generating novel ARV combinations with higher virulence and adaptation in chickens, which can evade existing immunity conferred by vaccines like S1133 [44,45].

Pathogenicity assessment in SPF chickens showed that the genotype VI strain was more virulent than the genotype III strain. Although both viral strains induced histopathological changes in tenosynovitis tissue characteristic of reovirus infection, genotype VI (G4) infection resulted in marked footpad swelling, a key hallmark of viral arthritis/synovitis, which was absent in the genotype III (WF) group and the uninfected controls. More critically, viral loads are significantly higher in key immune organs (spleen and bursa of Fabricius) of genotype VI (G4) infected group than that of genotype III (WF) group, which suggested that genotype VI (G4) has markedly higher pathogenicity compared to the genotype III (WF) strain. The pronounced replication in immune organs strongly suggests that G4, like other pathogenic ARVs, induces immunosuppression, compounding its direct pathogenic effects and increasing susceptibility to secondary infections in field [46]. However, a limitation of this study is the small sample size (*n* = 3 per group), which may have limited the ability to fully capture biological variability among different groups. Future studies involving larger and genetically diverse chicken populations are needed to further validate these findings.

In conclusion, this study confirms the widespread presence of antigenically variant and genetically diverse ARV strains (genotypes III and VI) in Chinese chicken flocks, capable of breaking through existing vaccine protection. The genotype VI strain (G4) exhibits significantly higher virulence than the genotype III strain (WF) tested, correlating with enhanced replication in vital organs and characteristic clinical signs. The detection of gene reassortment events underscores the dynamic evolution of these viruses. These findings collectively emphasize the critical and urgent need for the development and implementation of updated vaccines specifically tailored to combat the prevalent and highly pathogenic variant genotypes, particularly genotype VI, to mitigate the substantial economic losses caused by ARV in the Chinese poultry industry.

## Data Availability

The original contributions presented in this study are included in the article. Further inquiries can be directed to the corresponding authors.

## Supplementary Materials

The following supporting information can be downloaded at: https://www.mdpi.com/article/10.3390/microorganisms13112450/s1. Table S1: Primers used in the study.
