# Supplementary material for: Genetic and Pathogenic Characteristics of Variant Avian Reovirus Strains Isolated from Diseased Chickens in China
Source: Microorganisms. 2025 Oct 25;13(11):2450. doi: 10.3390/microorganisms13112450 (PMC12654140; doi:10.3390/microorganisms13112450)
Supplement: Supplementary file 1 [file microorganisms-13-02450-s001.zip › microorganisms-3789068-supplementary.pdf]

Table S1. Primers used in the study

| Target gene | Forward primer | Reverse primer | Size |
|---|---|---|---|
| ARV-L1 | TTCTCCGAACGCCGAAATGA | TCCAACGAGAGTCGACGCGATCA | 3882 bp |
| YV-L2 | ATGCATGTCAACGGGTTTGATGA | GCACGGACGCTACCTCGTTTCGG | 3780 bp |
| WF-L2 | ATGCATGTCAACGGGTTTGATGA | GAGTAATTCCTCGAGCCATGCCG | 3780 bp |
| G4-L2 | ATGCATGTCAACGGGTTTGATGA | GAGTAATTCCTCGAGCCATGCCG | 3780 bp |
| ARV-L3 | CCACCCATGGCTCAGATTAGAGGC | TACTGGAGGCCCGCGGTCATCTAG | 3858 bp |
| ARV-M1 | TTCTCGACATGGCCTATCTAGCC | ATCTCAAGACGACTAACCCAG | 2199 bp |
| YV-M2 | CCTTCGCTCTCAAGATGGGTAACG | GAGGCGATACCTCCTCAAGAGGG | 2031 bp |
| WF-M2 | CCTTCGCTCTCAAGATGGGTAACG | GAGGCGATACCTCCTCAAGAGGG | 2031 bp |
| G4-M2 | CCTTCGCTCTCAAGATGGGTAACG | GGCCTTACCACCTCAGGATGGTT | 2031 bp |
| ARV-M3 | GAGTCCTAGCGTGGATCATGGCG | GTGGGTACATGGAATCAGAGA | 1908 bp |
| YV-S1 | TTGTGTGCCGATGTTCCGTAT | TTARGTGTCGATGCCCGTACG | 1586 bp |
| WF-S1 | AGTCAATGTTCCGTATGTCCT | TTARGTGTCGATGCCCGTACG | 1586 bp |
| G4-S1 | AGTCAATGTTCCGTATGTCCT | TTARGTGTCGATGCCCGTACG | 1586 bp |
| ARV-S2 | TCTYCCACGATGGCGCGTGCCGT | GCAGCGTACGACCCTACGCCTAG | 1251 bp |
| ARV-S3 | AATGGAGGTACGTGTGCCAAA | TAACCGTCACATAGGTGGGAG | 1104 bp |
| ARV-S4 | TTGAGTCCTTGTGCAGCCATGGA | GAGGGTGTGGCGGCCCCTCACCC | 1104 bp |
